# Ex Vivo Partial Nephrectomy and Autotransplantation for Complex and Multifocal Renal Cell Carcinoma at a Single Institution: A Case Series

**DOI:** 10.7759/cureus.53686

**Published:** 2024-02-06

**Authors:** Ryu Kimura, Keiichiro Izumi, Kei Tanaka, Yoshinori Oshiro, Seiichi Saito

**Affiliations:** 1 Department of Urology, University of the Ryukyus, Nishihara, JPN; 2 Department of Urology, Cyubu Tokusyukai Hospital, Okinawa, JPN

**Keywords:** extracorporeal, renal tumor, partial nephrectomy, autologous, kidney transplantation

## Abstract

Renal autotransplantation is a rare surgical procedure designed to preserve renal function in patients with complex urinary system diseases or highly complex renal tumors. Between 2012 and 2023, four patients underwent ex vivo partial nephrectomy (PN) and autotransplantation for complex renal tumors at our hospital. Two patients had bilateral multifocal renal tumors, including von Hippel Lindau (VHL) disease and hybrid oncocytic chromophobe tumor (HOCT). The remaining two patients had highly complex renal tumors with Preoperative Aspects and Dimensions Used for an Anatomical (PADUA) score of 12, one of whom had a solitary kidney. None of the patients experienced any postoperative surgical complications. Pathologically, nine of the excised tumors had negative surgical margins, except for one of the four tumors on HOCT. Postoperative renal function decreased at one month compared to preoperative renal function (*P *= 0.01); however, there was no significant difference at three months (*P *= 0.07). None of the patients had a local recurrence or metastasis at the latest follow-up.Ex vivo PN and autotransplantation are feasible and reasonable treatment methods for highly complex and multifocal renal tumors regarding safety, local tumor control, and preservation of renal function.

## Introduction

Partial nephrectomy (PN) and radical nephrectomy (RN) are curative treatments for localized renal cell carcinoma [1]. Current guidelines recommend PN over RN for T1 renal tumors to preserve renal function [2]. In recent years, robot-assisted laparoscopic partial nephrectomy (RAPN) has become the preferred treatment for localized renal tumors [3]. RAPN is also used for complex renal tumors under appropriate expertise; however, outcomes for extremely complex tumors with a Preoperative Aspects and Dimensions Used for an Anatomical (PADUA) score of 12-13 are poor [4]. Ex vivo PN and autotransplantation are alternative approaches when conventional nephron-sparing surgery proves to be challenging [5,6]. We present four cases of highly complex renal tumors treated with ex vivo PN and autotransplantation.

## Case presentation

Case 1

**Figure 1:**
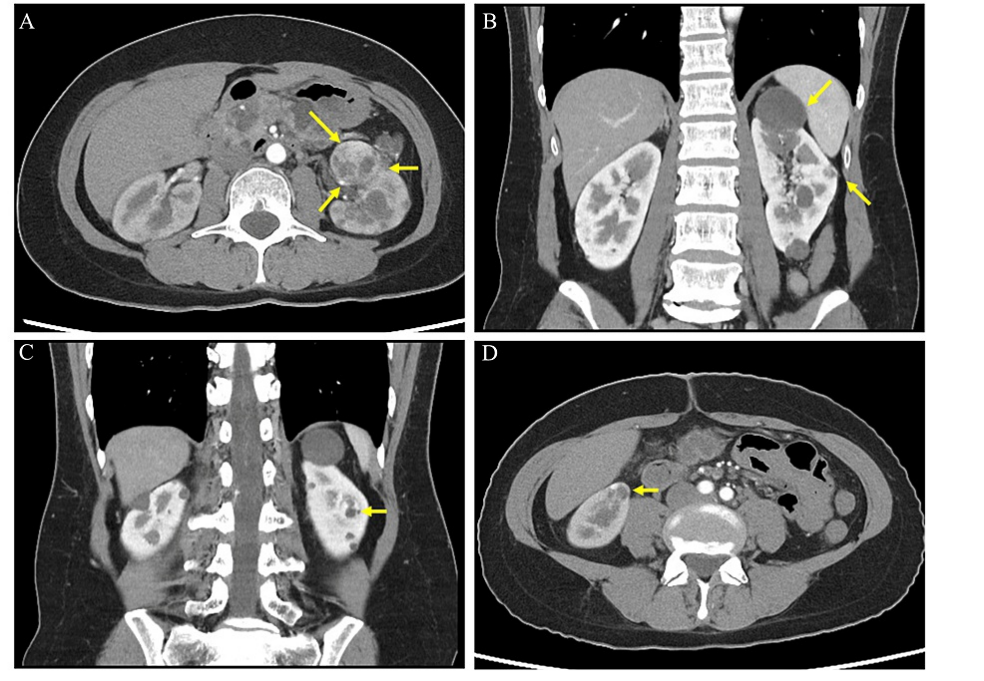
Bilateral multifocal tumors (maximum diameter 47 mm). (A) 36 mm renal tumor of the left kidney; (B) 48 and 16 mm renal tumors of the left kidney; (C) 18 mm renal tumor of the left kidney; (D) 17 mm renal tumor of the right kidney.

A 41-year-old woman was diagnosed with von Hippel Lindau (VHL) disease after an imaging test incidentally revealed left renal tumors. Computed tomography (CT) revealed four tumors with a maximum diameter of 48 mm in the left kidney and a 17 mm renal tumor in the right kidney (Figure 1).

**Figure 2:**
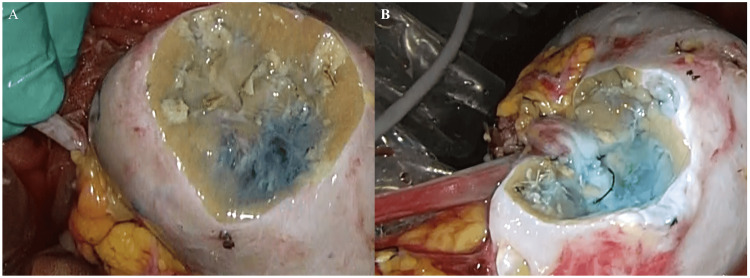
Perfusion of saline solution with methylene blue through (A) the renal artery to reveal the site of vascular injury and (B) the ureter to reveal the site of urinary tract injury.

Retroperitoneoscopic left kidney harvest and partial nephrectomy on a bench were performed. The kidney was perfused with Euro-Collins perfusate on crushed ice, and four tumors were carefully resected using ultrasound to confirm their location and depth. Following tumor resection, saline solution with indigo blue was irrigated from the renal artery and ureter to repair the site of the vascular and urinary tract injury (Figure 2).

Following tumor resection, the renal artery and vein were anastomosed to the ipsilateral external iliac artery and vein, and the ureter was anastomosed to the bladder using the Lich-Gregoir technique. She was discharged on the 11th day of hospitalization without postoperative complications. The pathology of the four renal tumors was clear cell renal cell carcinoma, pT1a-T1b, Fuhrman grade 1, and the resection margins were all negative. Thirteen months after surgery, the tumor has not recurred, and renal function has been maintained.

Case 2

**Figure 3:**
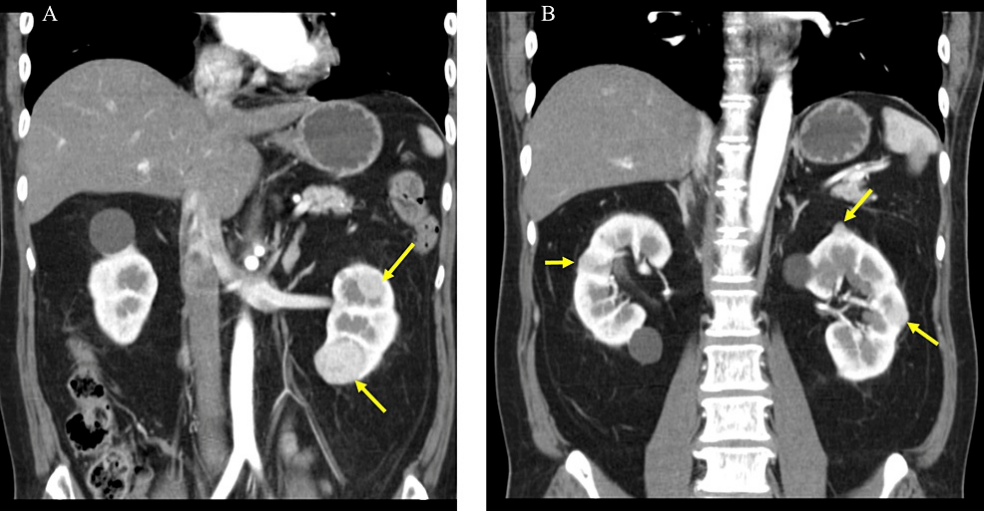
Bilateral multifocal tumors (maximum diameter 40 mm). (A) 40 and 26 mm renal tumors of the left kidney; (B) 12 mm renal tumor of the right kidney and 22 and 12 mm renal tumors of the left kidney.

A 55-year-old man with a history of myotonic dystrophy was referred to our hospital after a medical examination revealed a left renal tumor. CT showed four tumors with a maximum diameter of 40 mm in the left kidney and a 12 mm renal tumor in the right kidney (Figure 3).

Retroperitoneoscopic left kidney harvest and ex vivo nephrectomy were performed using the same procedure as in the previous case. The renal artery and vein were anastomosed to the ipsilateral external iliac artery and vein, and the ureter was anastomosed to the bladder using the Lich-Gregoir technique. He had no complications related to the surgical procedure but required rehabilitation due to comorbidities. The pathology of the four renal tumors was hybrid oncocytic chromophobe tumor (HOCT), pT1a. Grossly, the tumor appeared to have been completely resected; however, pathologically, one of the four tumors showed a positive margin. The patient was discharged 33 days postoperatively and has been free of recurrence at 6 months postoperatively.

Case 3

**Figure 4:**
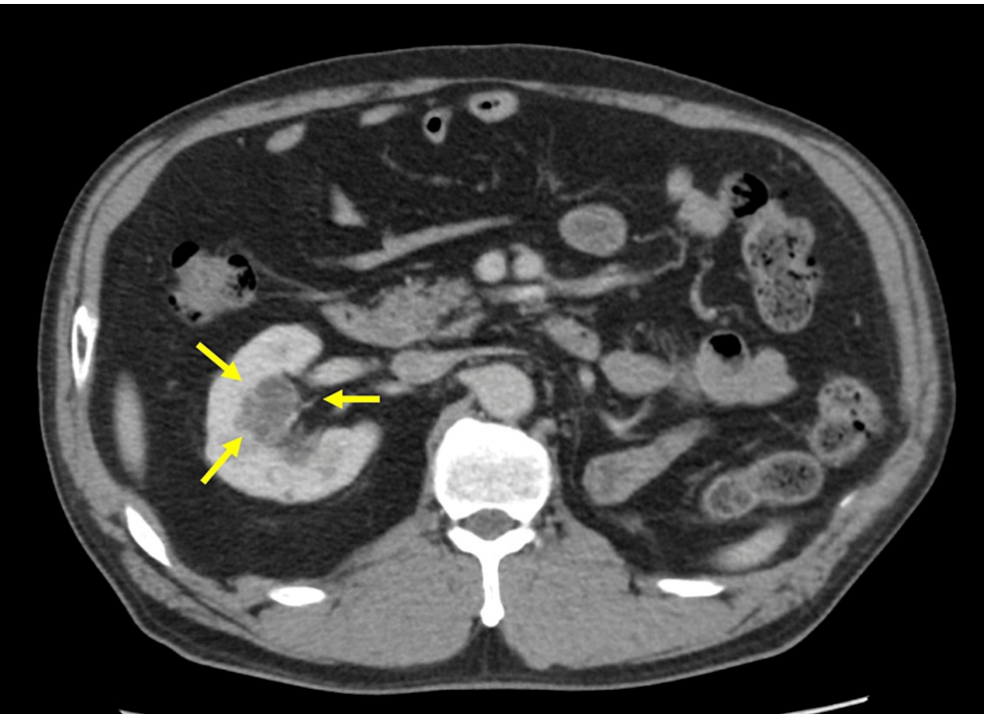
Completely embedded cystic tumor of the right kidney (27 mm).

A 62-year-old man who underwent laparoscopic left nephrectomy for T1b left renal cell carcinoma two years earlier showed a trend toward an enlarged right renal cystic lesion on imaging studies. CT showed a completely embedded 27 mm cystic tumor with a PADUA score of 12 in the right kidney (Figure 4).

Laparoscopic right kidney harvest and ex vivo partial nephrectomy were performed. The tumor was meticulously excised using an ultrasound guide. The resected tumor was submitted to rapid pathology and negative margin were confirmed. The kidney was subsequently anastomosed with the two renal arteries to the ipsilateral external and internal iliac arteries, and the renal vein was anastomosed to the external iliac vein. The ureter was anastomosed to the bladder with the Lich-Gregoir technique. The patient required four temporary dialysis treatments but was later weaned from dialysis and discharged after 31 days of hospitalization. Pathological examination revealed clear cell carcinoma, pT1a, Fuhrman grade 1, with a negative margin. Seven years after surgery, the tumor has not recurred, and renal function has been maintained.

 

Case 4

**Figure 5:**
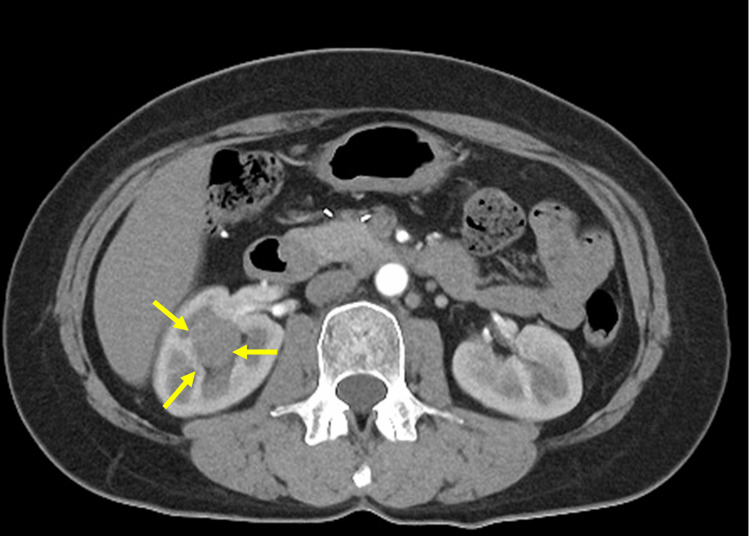
Completely embedded hypovascular tumor of the right kidney (25 mm).

A 53-year-old woman was referred to our hospital after an imaging study revealed an incidental right renal tumor. CT showed a completely embedded 25 mm tumor with a PADUA score of 12 in the right kidney (Figure 5).

Laparoscopic right kidney harvest and ex vivo partial nephrectomy were performed using the same procedure as in the previous case. The renal artery and vein were subsequently anastomosed to the ipsilateral external iliac artery and vein, and the ureter was anastomosed to the bladder using the Lich-Gregoir technique. The postoperative course was uneventful, and the patient was discharged on the 10th day of hospitalization. Pathology showed chromophobe cell renal carcinoma, pT1a, with a negative margin. The patient is alive and well, with no recurrence at the 14-month follow-up.

Postoperative changes in renal function

**Figure 6:**
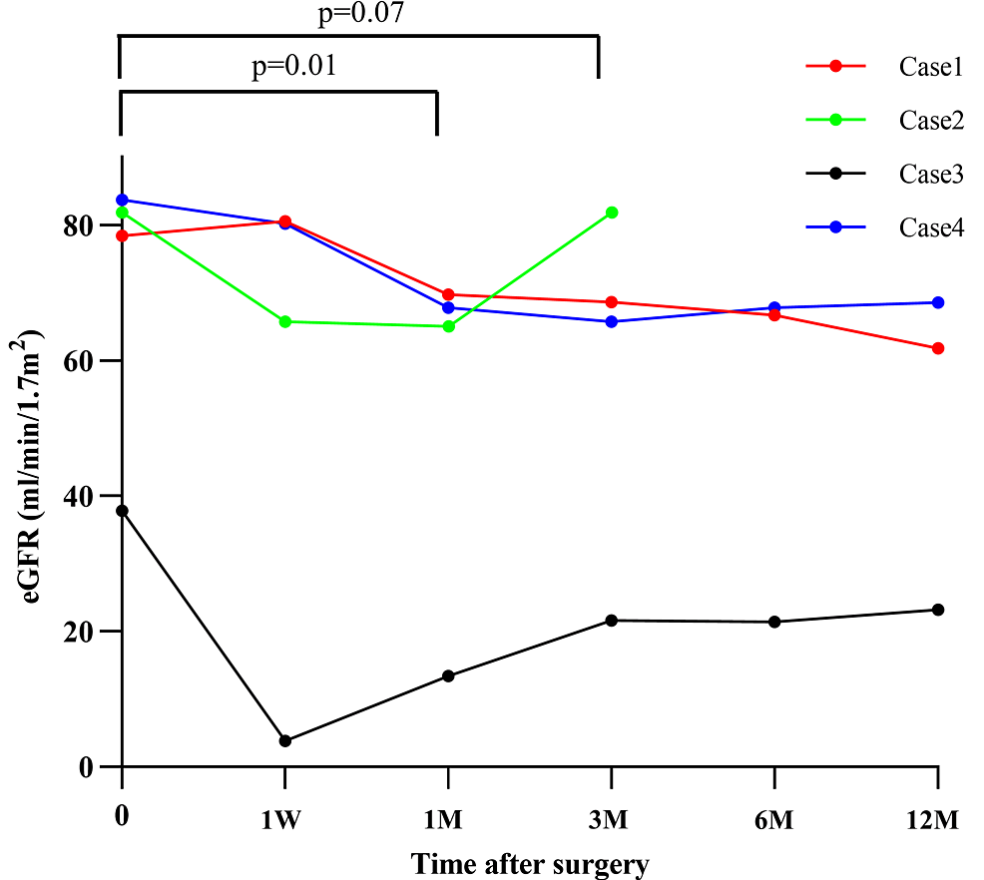
Changes in postoperative renal function in four cases. eGFR, estimated glomerular filtration rate

Postoperative changes in renal function in the four patients were compared with baseline renal function using a paired t-test implemented in JMP 20 software (SAS Institute Inc., Cary, NC). Figure 6 shows the postoperative changes in renal function in the four cases. Postoperative renal function decreased at one month compared to preoperative renal function (*P *= 0.01); however, there was no significant difference at three months (*P *= 0.07).

## Discussion

PN is the standard surgical procedure for T1a renal tumors. PN is currently recommended over RN for T1b renal tumors if technically feasible [2]. PN reduces cardiovascular morbidity and improves overall survival compared to RN [7,8]. RAPN has become the most common PN technique in the past decade and is often attempted in challenging cases [9]. RAPN can preserve renal function and local tumor control. However, RAPN for highly complex renal tumors has been shown to increase the probability of failure to achieve optimal surgical outcomes, even when performed by skilled surgeons [4]. In such patients with complex renal tumors, open partial nephrectomy is often performed to cool the kidney and preserve renal function, but the long ischemic time does not always lead to adequate preservation of renal function [10]. In terms of minimal decline in renal function and securing resection margins without complications, we indicated ex vivo PN and autotransplantation as alternative treatment options for such challenging cases. Renal autotransplantation is performed for complex renal vascular diseases (renal artery aneurysms), extensive ureteric injuries, and conventionally unresectable renal tumors [6,11,12]. Several studies have reported that ex vivo PN and autotransplantation for highly complex renal tumors in solitary or bilateral multifocal kidneys result in excellent tumor control and preservation of renal function [12-15].

We believe that the advantages of ex vivo PN and autotransplantation for highly challenging renal tumors are as follows: (1) the kidney is irrigated with cold perfusate and placed under extremely low temperatures on slush ice to preserve maximum renal function without concern for the ischemic time; (2) bench surgery allows for reliable tumor resection at a close range and under direct vision; and (3) perfusion of saline solution with methylene blue through the renal artery and ureter allows for reliable repair of vascular and urinary tract injuries. Our patients showed a decrease in renal function after one month compared with that preoperatively; however, at three months, renal function was maintained at the preoperative level. No major postoperative complications were observed. In this report, a positive surgical margin was observed in one of 10 tumors, but none of the cases had local recurrence at the latest follow-up. Although we did not submit intraoperative frozen pathology specimens in all cases to confirm negative margins, we believe that frozen pathology specimens will be necessary in the future to eliminate positive margins.

## Conclusions

In conclusion, we reported four cases of ex vivo PN and renal autotransplantation for highly challenging renal tumors. Ex vivo PN and autotransplantation are alternative approaches when conventional nephron-sparing surgery is challenging.

We found that ex vivo PN and autotransplantation are feasible and reasonable treatment choices for highly complex and multifocal renal cell carcinoma regarding safety, local tumor control, and preservation of renal function. These findings have important implications in light of the poor outcomes reported for RAPN in cases of complex renal tumors.
